# Genetic Diversity and Genome-Wide Association Study of Seed Aspect Ratio Using a High-Density SNP Array in Peanut (*Arachis hypogaea* L.)

**DOI:** 10.3390/genes12010002

**Published:** 2020-12-22

**Authors:** Kunyan Zou, Ki-Seung Kim, Kipoong Kim, Dongwoo Kang, Yu-Hyeon Park, Hokeun Sun, Bo-Keun Ha, Jungmin Ha, Tae-Hwan Jun

**Affiliations:** 1 Department of Plant Bioscience, Pusan National University, Miryang 50463, Korea; 601588zky@pusan.ac.kr (K.Z.); kk7ing@pusan.ac.kr (D.K.); eksvnd951@pusan.ac.kr (Y.-H.P.); 2 FarmHannong, Ltd., Nonsan 33010, Korea; leehan26@snu.ac.kr; 3 Department of Statistics, Pusan National University, Busan 46241, Korea; kkp7700@gmail.com (K.K.); hsun@pusan.ac.kr (H.S.); 4 Department of Applied Plant Science, Chonnam National University, Gwangju 61186, Korea; bkha@jnu.ac.kr; 5 Department of Plant Science, Gangneung-Wonju National University, Gangneung 25457, Korea; j.ha@gwnu.ac.kr; 6 Life and Industry Convergence Research Institute, Pusan National University, Miryang 50463, Korea

**Keywords:** peanut, core collection, genetic diversity, population structure, genome-wide association study, linkage disequilibrium

## Abstract

Peanut (*Arachis hypogaea* L.) is one of the important oil crops of the world. In this study, we aimed to evaluate the genetic diversity of 384 peanut germplasms including 100 Korean germplasms and 284 core collections from the United States Department of Agriculture (USDA) using an Axiom_Arachis array with 58K single-nucleotide polymorphisms (SNPs). We evaluated the evolutionary relationships among 384 peanut germplasms using a genome-wide association study (GWAS) of seed aspect ratio data processed by ImageJ software. In total, 14,030 filtered polymorphic SNPs were identified from the peanut 58K SNP array. We identified five SNPs with significant associations to seed aspect ratio on chromosomes Aradu.A09, Aradu.A10, Araip.B08, and Araip.B09. AX-177640219 on chromosome Araip.B08 was the most significantly associated marker in GAPIT and Regularization method. Phosphoenolpyruvate carboxylase (PEPC) was found among the eleven genes within a linkage disequilibrium (LD) of the significant SNPs on Araip.B08 and could have a strong causal effect in determining seed aspect ratio. The results of the present study provide information and methods that are useful for further genetic and genomic studies as well as molecular breeding programs in peanuts.

## 1. Introduction

### 1.1. Peanut Information

Peanut or groundnut (*Arachis hypogaea* L.) is an important oil and cash crop of the world [1]. Peanut seeds are rich in oil (48–50%) and protein (25–28%) and they contain certain vitamins and minerals which allows them to be used as an energy source for humans [2,3]. In addition, peanuts contain rich functional elements, such as oleic acid, linoleic acid, resveratrol, fiber, and vitamins [4,5,6].

Since the beginning of agriculture, food grains have been subjected to selection and breeding for size and most of the grains have seeds far larger than their wild relatives [7]. In the United States, peanut seed size is one of the standards used to determine the grade of shelled peanuts and to evaluate the commercial potential of advanced peanut breeding lines prior to the release of varieties [8]. 

There have been some studies on seed size in peanut. Quantitative trait loci (QTL) study were conducted to identify loci controlling seed size using a 142 backcross population (87 BC3F1 and 55 BC2F2) with two parents under two water regimes in peanut, while several QTLs associated with increased seed width were detected under water-limited treatment [9]. Simple sequence repeat (SSR) marker PM375 associated with seed length was identified in a total of 88 F2:6 recombinant inbred lines (RILs), representing that increase in seed length may influence in an increase in the weight of a hundred seeds, or in the length of the pod [10]. Florida-07 by GP-NC WS 16. A major seed size QTL on chromosome A05 was identified in the US peanut mini core collection using RILs from a cross between Florida-07 and GP-NC WS 16 [8]. However, there are few studies on seed shape in peanuts so far.

### 1.2. Peanut Germplasms and Core Collection

Various germplasms with large genetic diversity are excellent resources for peanut breeders to broaden the genetic basis of breeding materials and integrate important alleles related to valuable traits [11]. Diverse germplasms in peanuts have been used to enrich genetic resources, introduce resistance to diseases and pests and, finally, to improve the yield potential through continuous breeding programs.

Recently, effective methods to evaluate and introduce a genetic diversity of germplasm resources have been performed in various studies. Core collections were first defined as a limited set of accessions “representing, with a minimum of repetitiveness, the genetic diversity of a crop species and its wild relatives” [12,13]. The use of core collections has many advantages and they also represent a good starting material for association mapping. Recently, core collections have been established in various crops, including rice [14], wheat [15], maize [16], and *Brassica napus* [17]. The peanut core collections were developed from the US germplasm collection [18], and information on the accessions of the core collection are available at the Germplasm Resource Information Network (GRIN) (https://www.ars-grin.gov).

To promote and improve the utilization of germplasm resources in peanut breeding programs, the peanut mini core collection was established by utilizing the stratification strategy of the United States (US) peanut germplasm resource center [19]. The majority of the accessions in the mini core collection were unrelated individuals, which may be a good starting material for initiating the peanut association study. The purpose of establishing a core or mini core collection for any crop is to promote the efficient and economical use of plant materials by end-users and to identify germplasms with desirable characteristics. 

The Oil Crops Research Institute of the Chinese Academy of Agricultural Sciences in China established a core collection with 576 *A. hypogaea* genotypes and a mini core collection with 298 accessions representing the majority of the genetic diversity of cultivated peanut in China. They conducted an association study using the mini core collection, and a total of 89 simple sequence repeat (SSR) alleles were identified as associated with 15 agronomic traits. The results showed that there was a great possibility to combine association analysis and marker-assisted breeding using the peanut mini core collection [20,21]. The US mini core collection was evaluated and mapped using quantitative trait loci (QTL) for several traits, such as resistance to Tomato spotted wilt virus (TSWV) [21]. In the ICRISAT mini core collection, several candidate regions associated with non-redundant leaf proteins were identified as being related to tolerance to water deficit stress; however, little has been reported regarding these traits in the US germplasms [22].

### 1.3. Characteristic of Peanut Genome

Cultivated peanut is allotetraploid (2n = 4× = 40, AABB) with a genome size of 2800 Mb/1C and the genome composition of cultivated peanut was shown to have derived from a recent hybridization of *A. duranensis* (A subgenome) and *A. ipaensis* (B subgenome) [23,24,25,26]. As the polyploidization event occurred recently, the genetic diversity of cultivated peanut is extremely low [27]. Peanut subgenomes are very closely related [28,29] and have an estimated repetition rate of 64% [1], which makes the assembly of peanut genome sequences extremely difficult [1,26,30]. The genome sequences of the diploid ancestors (*A. duranensis* and *A. ipaensis*) of cultivated peanut were reported in 2016, which became the basis for understanding the genome of cultivated peanut [26]. The sequencing results of A. *duranensis* (A genomic progenitor) and A. *ipaensis* (B genome progenitor) provided new insights into the biology, evolution, and genome changes of cultivated peanut and accelerated the molecular breeding of peanut varieties [31].

Recently, the cultivated peanut allotetraploid A. *hypogaea* genome was sequenced in 2019 and compared with the related diploid *A. duranensis* and *A. ipaensis* genomes. A total of 39,888 A subgenome genes and 41,526 B subgenome genes were annotated in the allotetraploid subgenome [32].

### 1.4. Development of Molecular Markers Using Next Generation Sequencing (NGS) Technology

In 2005, pyrosequencing technology was implemented using large-scale parallel sequencing or deep sequencing, revolutionizing next generation sequencing (NGS) technology and biological genomic research [33]. In the past decade, NGS technology made significant progress, and the cost of sequencing dropped sharply [27]. In addition, there have been innovative improvements in the productivity and accuracy of sequencing data. In particular, genome-wide studies using de novo assembly, resequencing, and a variety of bioinformatic methods have enabled the production of large numbers of single-nucleotide polymorphisms (SNPs) and simple sequence repeats (SSR) in complex genomes [26,34,35,36]. In recent work, high-throughput genotyping was conducted using NGS technology through double-digest restriction-site-associated DNA sequencing (ddRADseq), a total of 14,663 SNPs were developed, and a genetic linkage map based on SNPs was constructed using 1765 SNP markers in 166 F9 RIL population from a cross between Zhonghua 5 and ICGV86699 [37]. Numerous SNP and cleaved amplified polymorphic sequence (CAPS) markers were developed from the re-sequencing of two Korean peanut germplasms of K-Ol and Pungan, which indicates that the molecular marker information can provide valuable guidance and information for peanut breeding programs [27].

Due to the relatively large genome size and the low genetic diversity in cultivated peanut, developing SNP array chips for high-throughput genotyping is necessary [38]. By DNA resequencing and the RNA sequencing of 41 peanut genetic materials and wild diploid ancestors, a total of 163,782 SNPS were obtained. A total of 58,233 unique SNP sequences with large amounts of information were selected to construct the high-density SNP array Axiom_Arachis with 58K SNPs [39]. The high-density SNP Axiom_Arachis array with 58K SNPs could be used to accelerate the process of high-resolution mapping and molecular breeding in peanuts. 

### 1.5. Applications of High-Density SNP Arrays in Crops

As the most abundant type of DNA sequence variation in the genome, SNPs could be successfully used to associate the genotypic variations with target phenotypes. High-density SNP arrays have been developed for high-resolution mapping of crops and are widely used in many applications that require a large number of molecular markers, such as high-density genetic profiling, genome-wide association study (GWAS), and genomic selection [38,40,41]. One hundred and seven U.S. peanut mini core collections were genotyped using a 58K Affymetrix SNP array and a total of 13,527 highly polymorphic SNP markers were selected for marker-trait associations in arachidic and behenic fatty acid compositions [42]. A total of 2882 polymorphic SNPs retained from the second edition of the Axiom_Arachis array (Axiom_Arachis2) were used to identify loci controlling pod construction trait using 195 F7 recombinant inbred lines (RILs) [43]. The 48K Axiom Arachis2 SNP array was applied to identify single nucleotide polymorphisms (SNP) among the two sets of RILs and the two original Nod+ parental lines to explore the genetic factors and genetic regions controlling nodulation in peanut [44].

Genomic-assisted breeding (GAB) using large amounts of genomic data related to important agronomic traits could be used to develop new varieties faster than when using traditional breeding methods. Detailed genetic maps consisting of thousands of array-based SNPs have been used for the identification of genes controlling target traits [41,45]. GWAS, also known as whole-genome association study, is an observational study of a genome-wide set of genetic variants in different individuals to investigate whether any variant is associated with the target traits [46]. Any phenotypic differences could then be connected back to the underlying causative loci via various mapping approaches, including quantitative trait loci (QTL) mapping. Many research groups have used GWAS to identify associations between genotypes and phenotypes as well as to discover novel biological mechanisms [47]. Currently, most GWAS have been performed using high-throughput SNP data obtained by SNP arrays with a greater density of variants and a wide range of allele frequencies [48,49,50,51]. The GWAS format is easy to share and generate, and GWAS can be conducted using various applications and software [46].

### 1.6. Purpose

In this study, we aimed to (1) evaluate the population structure and genetic diversity of 384 peanut germplasms including 100 Korean germplasms and 284 United States Department of Agriculture (USDA) core collections using Axiom_Arachis array with 58K SNPs, and (2) to conduct GWAS for seed shape and identify candidate genes associated with this trait. Our results could provide useful tools for improving various agronomic traits in molecular breeding programs for peanuts.

## 2. Materials and Methods

### 2.1. Plant Materials, DNA Extraction, and Genotyping

A total of 384 peanut accessions were used for the present study (Supplementary Table S1). Among those, 284 peanut accessions were obtained from the core collections of the US Department of Agriculture (USDA) according to the proportion of the number of germplasms, which were widely distributed in East Asia, South Asia, West Asia, East Africa, South Africa, West Africa, North America, South America, Europe, and the Australian continent. In addition, 100 peanut germplasms were obtained from the National Agrobiodiversity Center Korean, RURAL DEVELOPMENT ADMINISTRATION (RDA)-GenBank Information Center, South Korea, including landraces, breeding lines, and cultivars. A young leaf from each individual accession was collected to extract the genomic DNA. A total of 384 peanut genomic DNA were extracted for each accession using the cetyltrimethylammonium bromide (CTAB) protocol with slight modifications [52]. The quality and quantity of the extracted DNA were determined using a NanoDrop ND-1000 (Thermos Fisher Scientific Inc., Wilmington, DE, USA) and 1% agarose gel electrophoresis.

A high-density SNP array Axiom_Arachis with 58K SNPs was used to obtain the genotyping data [39]. Reference genome builds were acquired from arahy.Tifrunner.gnm1.KYV3 (https://www.ncbi.nlm.nih.gov/assembly) to serve as controls in the array design.

### 2.2. Screening of Seed Aspect Ratio

The seed aspect ratio data (Supplementary Table S2) were obtained by scanning seed images. The scanning images were processed by ImageJ 1.52a software (https://imagej.nih.gov/ij/notes.html) to generate phenotype data for the genome-wide association study. The seed aspect ratio was calculated as the seed major axis divided by the seed minor axis. Ten seeds per accession were scanned at the same time, and the seed aspect ratios of the ten seeds were averaged (Supplementary Figure S1). The phenotype data were analyzed using the R program to conduct a t-test and normal distribution in the accessions.

### 2.3. Population Structure Analysis

A principle coordinate analysis (PCoA) was conducted using the software GenAlEx V6.503 [53,54]. The population structure of 384 peanut accessions was evaluated by Structure v2.3.4 software (https://web.stanford.edu/group/pritchardlab/structure_software/release_versions/v2.3.4/html/structure.html) under the admixture model. We compared the structures following the same parameters with K-values ranging from 1 to 10, and 20,000 Markov chain Monte Carlo iterations after a burn-in period of 10,000 iterations were carried out for three independent runs per K value. To make a decision for the optimum number for K, the delta K (ΔK) method used the software online “harvester structure”.

### 2.4. Genome-Wide Association Analysis

We analyzed the SNPs in Axiom_Arachis with a 58K array of the cultivated peanut using R software analysis tools. In the present study, the GAPIT package of R software—was used to conduct GWAS, and the enriched compressed mixed linear model (ECMLM) was selected for the analysis of association between SNPs and the phenotype data of interest [55]. The cutoff for significant association was a false discovery rate (FDR) adjusted *p*-value of less than 0.05. Candidate genes covering significantly associated SNPs were selected from the PEANUTBASE website tool (https://www.peanutbase.org) within a 150 kb region upstream or downstream of peak SNPs according to the linkage disequilibrium (LD) decay results. 

### 2.5. Linkage Disequilibrium (LD) Analysis

We performed linkage disequilibrium analysis for all possible pairs of SNPs with a minor allele frequency (MAF) greater than 0.01 in a dataset. To determine the degree of resolution achieved in the association analysis, both the genome and chromosome-wide LDs were estimated [56].

LD blocks were viewed using Haploview4.2, which uses permutation tests to determine the *p*-values for each pairwise correlation. The LD decay was calculated with PopLDdecay [57,58]. The physical distance of the LD decay plot was determined based on the *D’* values and distances between each pair of SNPs on each chromosome using a nonlinear model [59].

The standard descriptive LD parameter D’ was estimated as previously described by [60,61]. The average D’ value was calculated for each chromosome using Haploview software [60].

### 2.6. Regularization Method

In human genome-wide association studies, regularization methods based on penalized likelihood are popular regarding their application to identify disease-related genes or genetic regions as they are computationally efficient when used in analysis of high-dimensional genomic data [62,63,64,65,66,67,68]. The penalized likelihood function using an elastic-net penalty is defined as

(1) $$Q\left(\beta \right)=-l\left(\beta \right)+\lambda \alpha \sum_{j=1}^{p}|\beta_{j}|+\lambda \left(1-\alpha \right)\sum_{j=1}^{p}\beta_{j}^{2}$$

where l(β) is a log-likelihood function, β is the *p*-dimensional coefficient vector, λ≥0 is a tuning parameter for sparsity, and α ∈ [0,1] is a tuning parameter for smoothness. When α = 1, the coefficient vector *β* becomes the solution of the least absolute and shrinkage selection operator (LASSO) [69]. The estimated coefficient β consists mostly of zero values and only a few nonzero values. Based on 100 bootstrap samples, the selection probability of individual SNPs was computed where only SNPs with nonzero coefficients were selected for each bootstrap sample. Finally, we were able to identify the top ranked SNPs by their selection probability.

In order to select significant SNPs, we used two types of threshold of selection probability which can control the number of falsely selected SNPs. The first one is the theoretical threshold proposed by [70]. The second one is the empirical threshold [71] which basically computes the quantile value of an empirical distribution of selection probability based on permutation. In their extensive simulation studies, it was demonstrated that the number of falsely selected SNPs can be controlled when the empirical threshold is applied to high-dimensional genomic data. The theoretical threshold $\left(\pi_{\theta }\right)$ and the empirical threshold $\left(\pi_{\theta }^{*}\right)$ can be written as:

(2) $$\pi_{\theta }=\frac{q_{\Lambda }^{2}\;}{2\theta p}+\frac{1}{2}{\;\mathrm{and}\;\pi }_{\theta }^{*}=\frac{1}{B}\overset{B}{\underset{b=1}{\sum }}SP_{\left(b\right)}^{\left[\theta \right]}\left(I_{b}\right)\;,$$

where θ is the upper bound of the expected number of false discoveries, $q_{\Lambda }$ is the average number of selected SNPs, B is the number of permutations and $I_{b}$ is the b-th random permuted sample. We denote $SP_{\left(b\right)}^{\left[\theta \right]}$ by the top θ-th ranked selection probability when they were sorted in descending order for the b-th permuted sample such as $SP_{\left(b\right)}^{\left[1\right]}>\cdots >SP_{\left(b\right)}^{\left[p\right]}$. We chose the expected number of false discoveries θ=1, and thereby the number of falsely selected SNPs by each threshold can be guaranteed to be less than θ=1.

## 3. Results

### 3.1. SNP Genotyping

**Figure 1:**
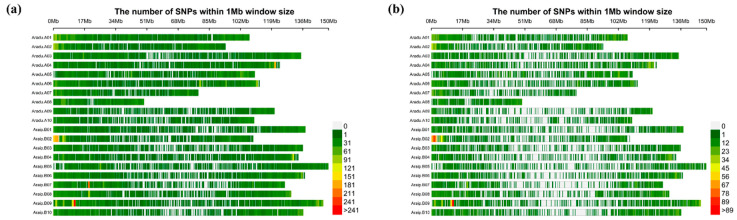
Single nucleotide polymorphisms (SNP) distribution in the 20 chromosomes of the cultivated peanut. The horizontal axis shows chromosome length (Mb), the shades of red represent SNP density. The vertical axis shows the 20 chromosomes. (**a**) Polymorphic SNPs except for scaffold markers; (**b**) Polymorphic SNPs (except for scaffold markers) after filter by GAPIT coding.

Of the 58K informative SNPs, a total of 47,837 polymorphic SNPs were selected (Supplementary Table S3). Of the 47,837 SNPs, 19,554 and 21,876 SNPs were derived from the subgenomes A and B, respectively, and 6407 SNPs were derived from scaffolds (Supplementary Table S3 and Figure 1a). A total of 14,030 SNPs were selected for association analysis after eliminating SNPs with high levels of missing data (>20%), heterozygosity (>20%), or low a minor allele frequency (MAF) (<0.01). Of the 14030 SNPs, 6623 and 7407 SNPs were derived from A and B subgenomes, respectively. The majority of SNPs were evenly distributed across the chromosomes; however, there were some large gaps between SNPs on the chromosomes Aradu.A09, Aradu.A10, Araip.B05, Araip.B06, Araip.B07, Araip.B09, and Araip.B10 (Figure 1b). The peanut genome had an overall SNP density of 5.91 SNPs/Mb, with the Aradu.A09 (3.45 SNPs/Mb) and Aradu.A08 (9.35 SNPs/Mb) chromosomal densities being the lowest and highest, respectively (Supplementary Table S4).

### 3.2. Phenotype Data Analysis

**Figure 2:**
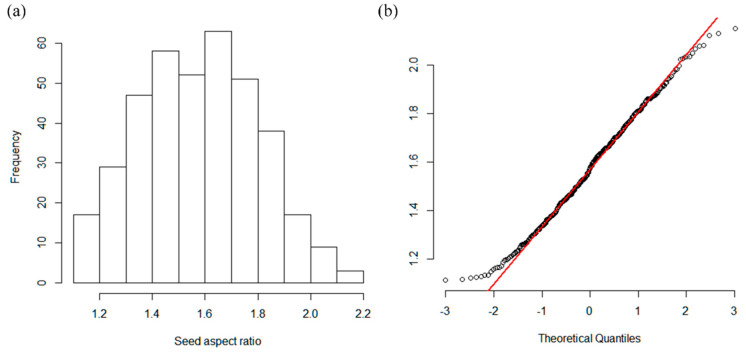
(**a**) Peanut seed aspect ratio data histogram; (**b**) The normal distribution test by the quantile-quantile (QQ plot) graph.

The mean value for the seed aspect ratio was 1.6325 (Figure 2a). The normal distribution test showed that the scatter points of the quantile–quantile (QQ plot) graph (Figure 2b) were clustered around the fixed line; therefore, we assumed that the data were normally distributed (*p* = 0.05).

### 3.3. Genetic Diversity

**Figure 3:**
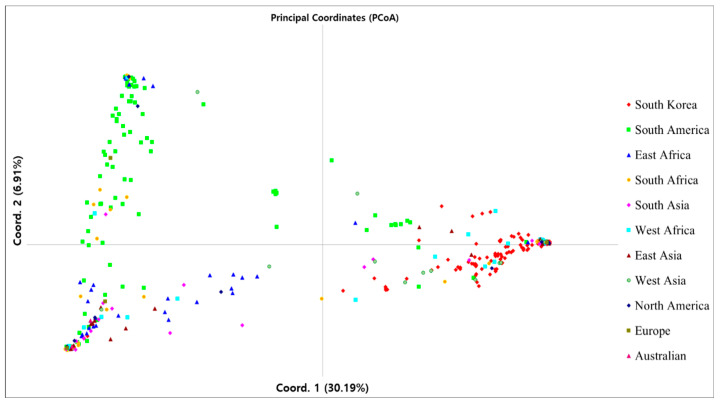
The pattern of Principal Coordinates Analysis (PCoA).

The pattern of PCoA (Figure 3) showed that the first two axes accounted for 30.19% and 6.91%, respectively, of the total variation and the 384 peanut accessions were divided into three broad groups across the first two axes. The first axes separated the South Korean (clustered filled diamonds) and South American (green filled squares) peanut accessions into two very different parts, and, at the same time, assigned East Asian, South Asian, and West Asian peanut accessions (brown filled triangles, pink filled diamonds, and green filled circles, respectively) to another part. Additionally, the peanut accessions that originated from East Africa, South Africa, West Africa, North America, and Europe formed two concentrated groups by the first and second axes. Interestingly, the accessions from South Korea were genetically very different from those from South America, which is the origin of the cultivated peanut.

### 3.4. Genetic Structure

**Figure 4:**
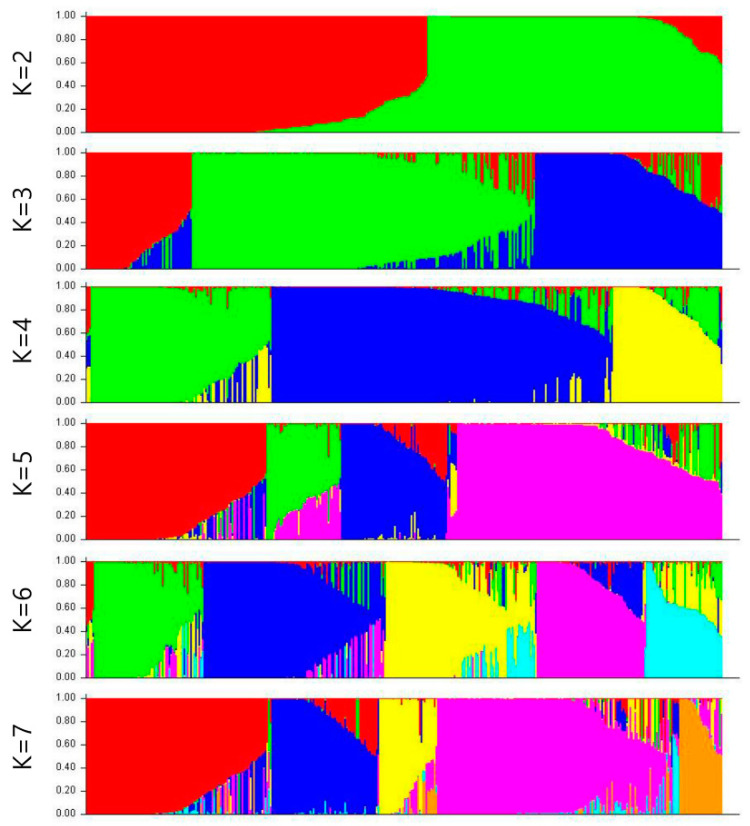
Structure clustering results obtained at K = 2 to K = 7 of the 384 peanut accessions. Each individual is represented by a bar corresponding to the sum of assignment probabilities to the K cluster.

At K = 2, we found maximum Δk values that were plotted against the K to confirm the number of populations, while another lower peak was shown at K = 7 (Supplementary Figure S2). When most individuals were divided into the two subpopulations (K = 2, Figure 4), the peanut accessions, including 64.9% from Asia (of which approximately 74% individuals were from South Korea and 26% from other origins in Asia), 24.4% from Africa, 10.2% from South America, and 0.5% from Europe, belonged to one subgroup (red), while another subgroup (green) revealed features of accessions, including 16.8% from Asia (comprising about 6.7% from South Korea and 93.3% from other Asia origins), 35.2% from Africa, 42.5% from South America, 2.8% from North America, 1.7% from Europe, and 1% from Australia. 

As we continued to divide the subgroups carefully, there were new divisions into the subgroups. The most divergent subgroups were formed at K = 7. Of the peanut accessions, 26.4% originating from Asia (of which approximately 10.3% were from South Korea and 89.7% from other Asia origins), 45.5% from Africa, 20.9% from South America, 3.6% from North America, 1.8% from Europe, and 1.8% from Australia belonged to the red subgroup. The green subgroup revealed features of 50% accessions from South Korea and 50% from Africa. The dark blue subgroup showed features of 1.5% accessions from Asia, 20% from Africa, 75.5% from South America, 1.5% from North America, and 1.5% from Europe. The yellow subgroup showed features of 91.4% accessions from Asia (including about 87.5% from South Korea and 12.5% from other Asia origins), 2.9% from Africa, and 5.7% from South America. The pink subgroup consisted of 56.3% accessions from Asia (of which approximately 63.8% were from South Korea, and 36.2% from other Asia origins), 31% from Africa, 12% from South America, and 0.7% from Europe. The light blue subgroup showed features of accessions from only South America. The orange subgroup showed features of individuals with 76.9% of accessions from Asia (of which approximately 85% accessions were from South Korea and 15% from other Asia origins), 15.4% from Africa, and 7.7% from South America.

### 3.5. Genome-Wide Association Study (GWAS)

**Figure 5:**
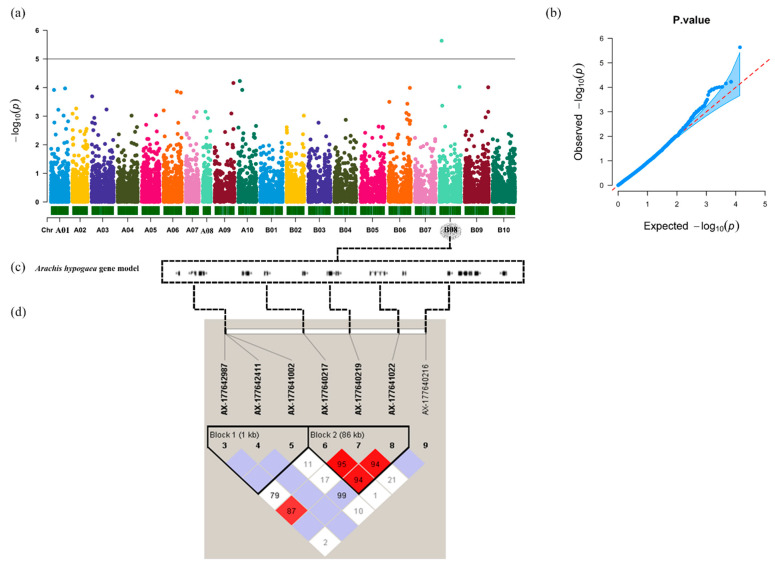
(**a**) Manhattan plot of a genome-wide association analysis by GAPIT; (**b**) Q-Q (quantile-quantile) plot; (**c**) Genes located at the association regions based on the *Arachis hypogaea* Tifrunner 1.0 reference genome; (**d**) Linkage disequilibrium (LD) plot generated using Haploview, D’ values that correspond to SNP pairs are shown within the respective squares. Higher D’ values are indicated with a brighter red color.

The genotype data of 14,030 filtered polymorphic SNPs and the phenotypic data of the seed aspect ratios were analyzed for GWAS by GAPIT. A total of five candidate SNPs showing significant associations (*p* < 0.0001) with the seed aspect ratio were identified on chromosomes Aradu.A09, Aradu.A10, Araip.B08, and Araip.B09 (Table 1 and Figure 5a). The distribution of the observed −log10(p) for each SNP was compared with the expected distribution in the QQ plot representing that the population structure and kinship relationship were well controlled in the GWAS (Figure 5b). The significance of the marker–trait associations were determined using the FDR with adjusted *p*-value (*p* = 0.05). AX-177640219 on chromosome Araip.B08 was significantly associated with the seed trait at the significant threshold (Table 1).

### 3.6. LD and Candidate Genes Analysis

Pairwise comparisons were performed between all SNPs for the estimation of LD decay. At a cutoff value of r^2^ = 0.1, the averaged LD decay distance of the 384 peanuts was approximately 150 kb (Supplementary Figure S3). The pattern of LD across the entire genome presented a number of haplotype blocks containing SNPs that can be used to determine the range of the candidate gene. The genomic locations harboring significant SNPs from the GWAS were investigated to identify putative candidate genes based on the peanut reference genome (*A. hypogaea* Tifrunner 1.0). Strong and extensive pairwise LD was observed among highly significant SNPs around AX-177640219 (*p*-value = 0.000015) on chromosome Araip.B08 from the 12,629,161 to 13,029,161 bp region (*D’* > 0.80) in which *D’* varied from 0.036 to 1 (Figure 5d and Supplementary Table S5).

Fifteen annotated genes at the association regions flanked by SNP AX-177640219 on chromosome Araip.B08 were identified within the estimated ±150 kb window based on the reference genome (Figure 5c and Supplementary Table S6).

### 3.7. Regularization Method

**Figure 6:**
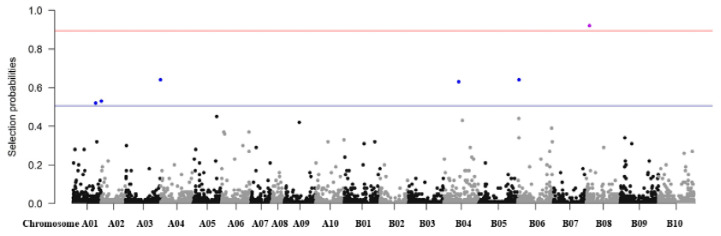
Manhattan plot of a genome-wide association analysis by the least absolute and shrinkage selection operator (LASSO).

Alternatively, we also conducted regularization methods, such as LASSO, to identify candidate regions associated with the seed aspect ratio (Figure 6) [71]. The regularization method was performed using an entire dataset at a time and could select several putative markers most likely related to the trait based on the value of selection probability, whereas the ECMLM analysis only tested one marker at a time. As a result, one SNP locus (AX-177640219 on Araip.B08) was identified as being most likely related to the seed aspect ratio based on the selection probability at the permuted threshold 0.894, and was also found to be highly significantly associated in the GAPIT analysis (Figure 6). When loosening the strict threshold to 0.506, a total of six SNPs were additionally identified, AX-177640938 on chromosome Araip.B08, AX-147218661 on Aradu.A03, AX-147251864 on Araip.B06, AX-176802342 on Araip.B04, AX-176791478 on Aradu.A02, and AX-176800768 on Aradu.A01, which presented significant associations with ECMLM results indicating that the regions flanked with these markers might be candidate regions for possible determination of seed shape in peanuts. Therefore, the use of both methods to conduct association studies is beneficial in (1) boosting confidence in the case where common markers are identified and (2) to maximize the possibility of finding new significant markers associated with a trait of interest.

### 3.8. Evaluation of Heterozygous Rate

**Figure 7:**
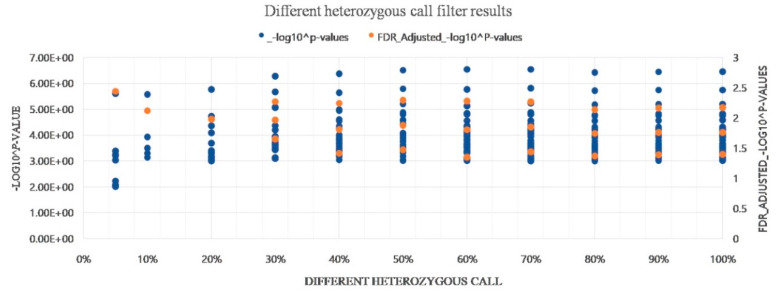
Different heterozygosity rates to filter genotype data and different significance cut-off thresholds used to assess the effect of the SNPs on seed aspect ratio.

The same filtering conditions with maximum missing data of 20% and MAF of 0.01%, different heterozygosity rates (starting from 5% and 10% and every 10% until 100% maximum heterozygous SNPs (Figure 7 and Supplementary Table S7) were used to filter the genotype data in our study, and different significance cutoff thresholds were used to assess the effect of the SNPs on the seed aspect ratio.

When the genotype data filtered by a 5% to 20% maximum heterozygous rate were used for GWAS analysis, a higher specificity of the results was obtained; however, only one significance marker was evaluated at the 0.05 critical threshold for the false discovery rate (FDR) adjusted p-value. On the contrary, when a high heterozygosity rate of 30% to 100% was used for data filtering, additional significant markers were detected; however, those markers require validation.

## 4. Discussion

The trait of seed ratio (length-width ratio) screened in this study has been reported to have very high broad-sense of heritability in recently published peanut research. Zhang et al. [72] reported that it has a high broad-sense of heritability (0.81) in peanuts. For other legume crops, Hu et al. [73] reported a very high broad-sense of heritability ranged from 92.46 to96.25 in three traits related to seed shape in soybeans. If a phenotypic trait has a high level of heritability, the influence of the environmental factors might be relatively small, and in this case, it could be possible that genes (or QTL) with relatively large effects on the trait could be identified even if the trait were not measured in the same conditions. Of course, even in this case, the influence of the environmental conditions cannot be overlooked.

The genotyping data from the 58K SNP array chip could play an important role in understanding the evolutionary history of peanuts and the domestication of cultivated peanut [74]. The application of the array chip also demonstrated that it is a powerful and reliable tool for peanut germplasm background selection and evolutionary studies [75]. In the present study, it is the first to conduct GWAS analysis using a large number of Korean peanut germplasms as well as the USDA peanut core collection with a high-density SNP chip data that can be used toward increasing the genetic diversity of the US peanut germplasm collection.

The cultivated peanut species (A. *hypogaea*) is known to originate from southern Bolivia to northwestern Argentina based on the occurrence of the two progenitor species, A. *duranensis* and A. *ipaensis*, and archaeological evidence gathered in those regions [76,77,78]. Researchers also suggested that the eastern slopes of Cordillera may be a possible area for the origin of A. *hypogaea* due to the favorable environment for peanut growth [78,79]. However, the present study showed an interesting result in that South American peanuts, generally regarded as the origin of peanuts, were revealed as having significant genetic differences from peanuts of other regions, including South Korea. 

The evaluation results for the evolutionary relationships among the entirety of the 384 peanut germplasms indicated that most of the peanut individuals from South Korea and South America separated into two distinct groups and were also independent from the peanuts from the other origins. This might indicate that there was a great genetic difference between the peanut germplasms from South Korea and South America. Likely, due to the lack of interactions between South Korean peanut germplasms and others, it might be possible that an independent breeding history by human selection and/or environmental influences for a long period have caused these genetic differences. 

In human genetic association studies with high-dimensional genomic data, regularization methods, such as LASSO and elastic-net, have been widely applied to identify outcome-related genetic sites and genes as they have certain advantages over univariate analysis. First, regularization methods can easily handle highly correlated genomic measurements and covariate effects as they are based on a regression model. Secondly, the majority of regularization methods have been implemented into very efficient computational algorithms such R package ‘glmnet’ and ‘gglasso’. These packages can detect outcome-related genetic-sites and genes in less than a minute for more than 100K dimensional genomic data. Lastly, there are various types of regularization methods that can be applied to different types of genomic data. For example, we applied LASSO and elastic-net to SNP data in the GWAS or QTL analysis; however, sparse group LASSO [80] and network-based regularization [81] are ideal for group structured genomic data, such as gene expression data and DNA methylation data. Despite these advantages of regularization methods, they have rarely been applied to detect QTLs or genes of interest in crops. In this study, LASSO was able to identify potentially outcome-related SNPs that were not identified in general GWAS methods although further validation studies are required for these SNPs.

Data filtering is the primary process of genome-wide association analysis, which includes huge amounts of data and requires strict quality control standards. Data filtering is divided into two sections, one for marker variables and another for individuals. The former considers the minor allele frequency (MAF) and the degree of missing data and heterozygosity, etc., whereas the latter mostly considers missing levels, population stratification, and independency among individuals [82]. The entire set of heterozygous SNPs are typically used in human GWAS analysis [83]. In peanuts, a high level of heterozygosity may not be expected as peanut is a self-pollinating crop revealed to have a low outcrossing rate ranging from 1.9% to 8% [84]. However, our array chip data showed a large number of heterozygous SNPs, which can affect the GWAS results. According to Figure 7, the significant SNPs identified from using 5% to 20% maximum heterozygous rate showed the same GWAS results, with one significant marker at FDR 0.05, while the results from using 30% to 100% maximum heterozygous rate showed similar GWAS results with three significant markers. Therefore, we filtered the genotype data with maximum heterozygous SNPs of 20%, and we used less heterozygous SNPs for analysis. 

Carbon assimilated by photosynthesis is transported into seeds with multiple purposes, such as the biosynthesis of starch, oil, amino acids, and cellulose. The most important aspect of oil accumulation in developing seeds lies in the activation of metabolic pathways driving incoming carbon into fatty acid biosynthesis at the expense of competitive pathways. Within the genomic region of ~300 kb associated with seed development, phosphoenolpyruvate (PEP) carboxylase (PEPC; Arahy.HT9EWH) was among eleven genes located within the LD of significant SNPs on the chromosome Araip.B08 (Supplementary Table S6). PEP is catalyzed into oxaloacetate (OAA), a protein precursor, by PEPC [85]. OAA can be converted to malate and then to pyruvate (a precursor for oil). PEPC had been reported to regulate the metabolic network of glycolytic carbon into precursors for both oil and protein in soybean seed development [86]. The activation status of PEPC has been reported to play a key role in the partitioning of assimilates into the different storage products in barley (*Hordeum vulgare*), alfalfa (*Medicago sativa*), and fava bean (*Vicia faba*) [87,88,89]. In peanuts, researchers reported that the expression levels of PEPC genes were significantly associated with lipid accumulation [90]. In the present study, only fifteen annotated genes were identified within the genomic region as being highly associated with seed development through high-throughput GWAS analysis. Among them, the PEPC gene could have a strong causal effect within this region associated with diverse metabolic pathways that includes including protein and oil biosynthesis.

## 5. Conclusions

Peanut is one of the most important food/oil crops and improving the quality and yield potential of crops is an important challenge in most breeding programs. Our study demonstrated the feasibility of GWAS analysis using the core germplasm from diverse origins and high-density array chips. Five candidate markers with a significant correlation with the aspect ratio of peanut seeds were identified and lay a foundation for further research. The Arahy.HT9EWH, phosphoenolpyruvate carboxylase (PEPC) gene corresponding to the most significantly associated marker was a promising candidate gene that is involved in many metabolic pathways, including those involved in seed development processes. Therefore, the results of the present study provide valuable information and methods for the genetic and genomic study as well as molecular breeding programs in peanuts.

## Figures and Tables

**Table 1:** Significant markers associated with seed aspect ratio of peanut identified using GAPIT analysis.

SNP	Chromosome	Position (bp)	*p*-Value (*p*)	FDR_Adjusted_*p*-Values
AX-177640219	Araip.B08	12829161	2.31 × 10^−6^	0.032
AX-147235444	Aradu.A10	8911644	5.91 × 10^−5^	NS ^a^
AX-176807953	Aradu.A09	113907685	6.95 × 10^−5^	NS
AX-176822392	Araip.B08	121783058	9.55 × 10^−5^	NS
AX-147262340	Araip.B09	143554366	9.80 × 10^−5^	NS

^a^ FDR_adjusted_*p*-value is not significant at the level of 0.05.

## Data Availability

Please refer to suggested Data Availability Statements in section “MDPI Research Data Policies” at https://www.mdpi.com/ethics.

## Supplementary Materials

The following are available online at https://www.mdpi.com/2073-4425/12/1/2/s1, Figure S1: ImageJ protocol, Figure S2: Δk values to confirm the number of populations, Figure S3: The estimation of LD decay, Table S1: Information on the 384 peanut accessions, Table S2: Information on the 384 peanut accessions seed aspect ratio, Table S3: A high-density SNP array ‘Axiom_Arachis’ with 58K SNPs, Table S4: Information of the peanut genome from 58K SNP array chip, Table S5: Pairwise LD on chromosome Araip.B08, Table S6: List of genes in the significant region (chromosome Araip.B08) with annotations identified by *Arachis hypogaea* Tifrunner 1.0 reference, Table S7: Genome-wide association study (GWAS) (*p*-value < 0.001) of seed aspect ratio were constructed by different heterozygous filter methods.
