# Supplementary figures and images for: Genetic Diversity and Genome-Wide Association Study of Seed Aspect Ratio Using a High-Density SNP Array in Peanut (Arachis hypogaea L.)
Source: Genes (Basel). 2020 Dec 22;12(1):2. doi: 10.3390/genes12010002 (PMC7822046; doi:10.3390/genes12010002)

## Slide 1
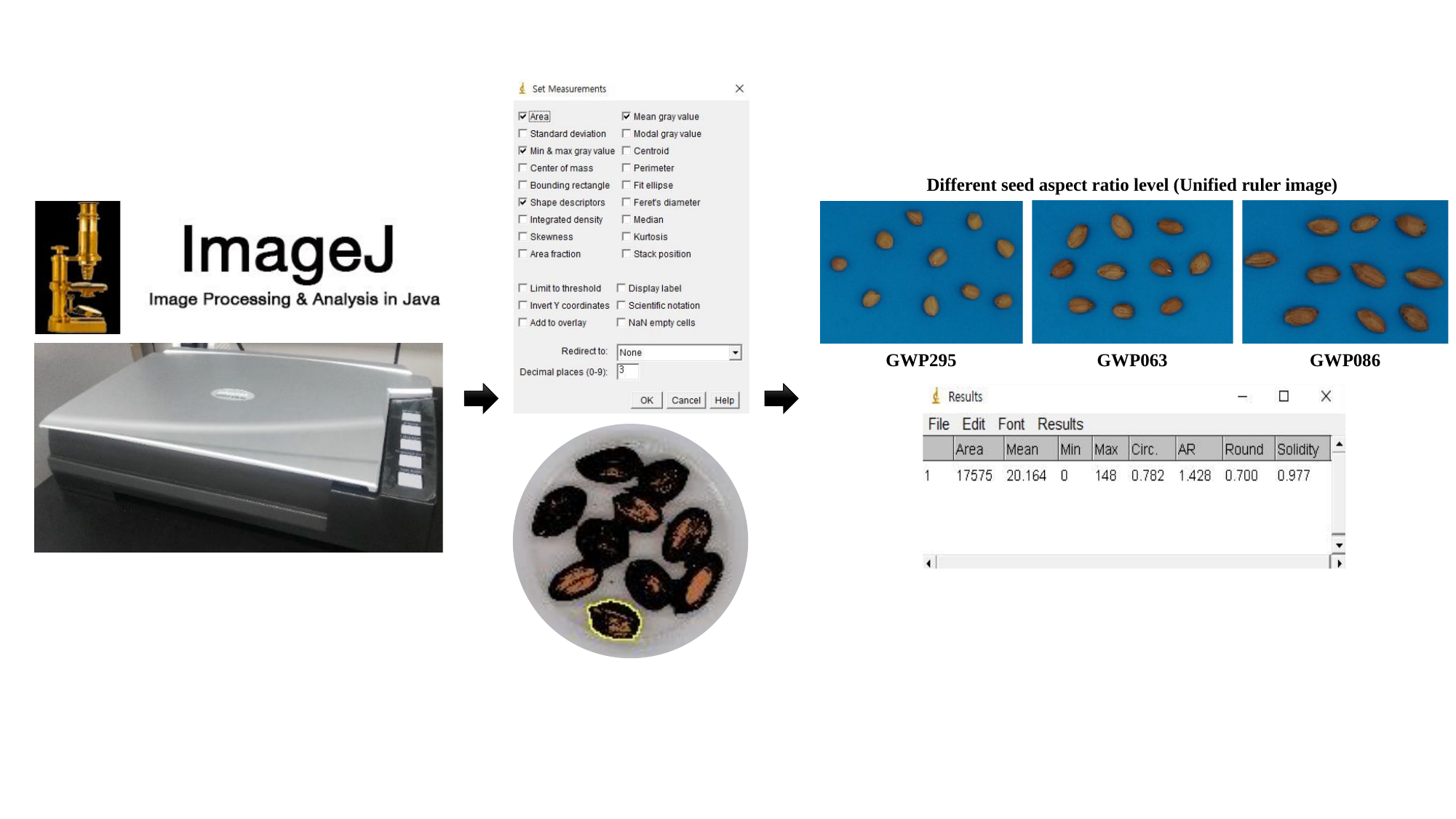

Different seed aspect ratio level (Unified ruler image)
GWP086
GWP295
GWP063

Supplement: Supplementary file 1 [file genes-12-00002-s001.zip › Supplementary Figure S1_final.pptx]

## Slide 1
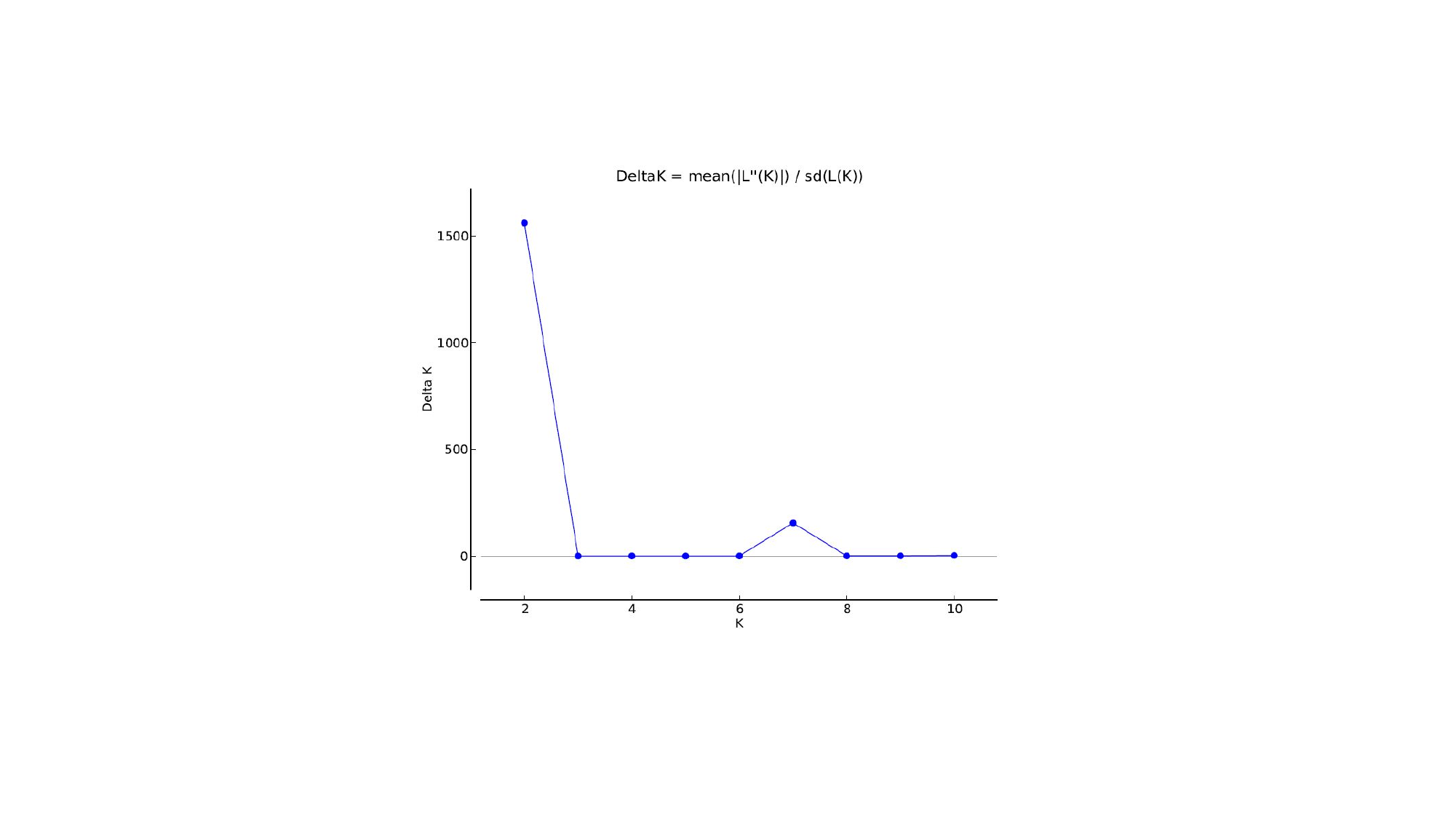

Supplement: Supplementary file 1 [file genes-12-00002-s001.zip › Supplementary Figure S2_final.pptx]

## Slide 1
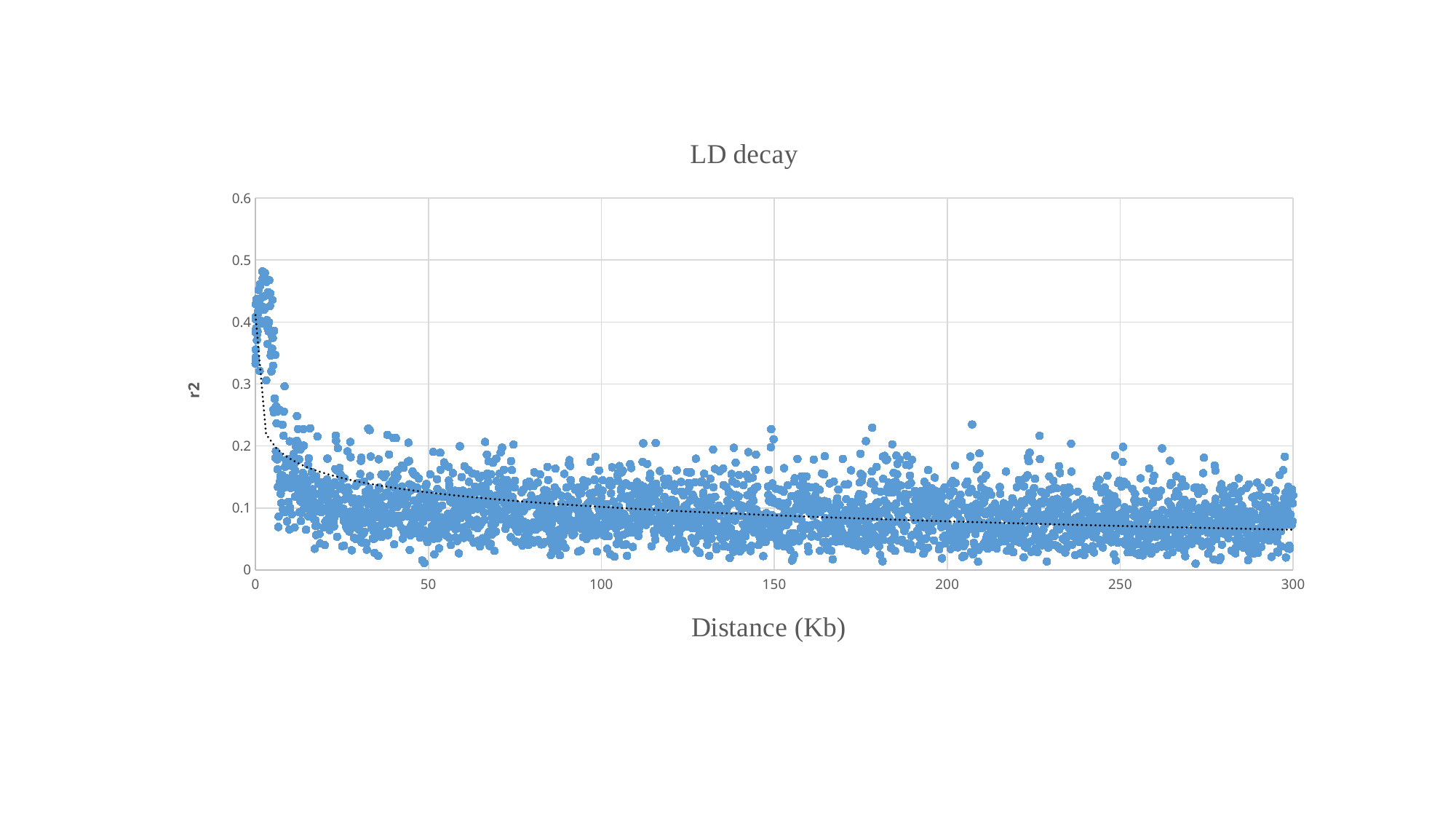

### Chart: LD decay
| Category | Mean_r^2 |
|---|---|

Supplement: Supplementary file 1 [file genes-12-00002-s001.zip › Supplementary Figure S3_final.pptx]
